# Prevalence and sociodemographic correlates of antinuclear antibody testing by indirect immunofluorescence or solid-phase assays in a Spanish population: the Camargo Cohort

**DOI:** 10.1007/s12026-023-09430-z

**Published:** 2023-11-04

**Authors:** Juan Irure-Ventura, Daniel Martínez-Revuelta, Marcos López-Hoyos, Marta Martín-Millán, Daniel Nan, Emilio Pariente, Javier Pardo-Lledías, Alejandra Comins-Boo, José Manuel Olmos, Víctor Manuel Martínez-Taboada, José Luis Hernández

**Affiliations:** 1 Immunology Department, University Hospital Marqués de Valdecilla, 39008 Santander, Spain; 2 Immunopathology Group, Marqués de Valdecilla University Hospital-IDIVAL, 39011 Santander, Spain; 3 Family Medicine. Healthcare center Astillero, Santander, Spain; 4 University of Cantabria, 39011 Santander, Spain; 5 Internal Medicine Department, University Hospital Marqués de Valdecilla, 39008 Santander, Spain; 6 Rheumatology Division, University Hospital Marqués de Valdecilla, 39008 Santander, Spain

**Keywords:** Antinuclear antibodies (ANA), Autoimmune disease (AID), Indirect immunofluorescence (IIF), Solid phase assays (SPA), Pre-test probability, Sociodemographic and biobehavioral features

## Abstract

Autoantibodies are the hallmark of autoimmunity, and specifically, antinuclear antibodies (ANA) are one of the most relevant antibodies present in systemic autoimmune diseases (AID). In the present study, we evaluate the relationship between ANA and sociodemographic and biobehavioral factors in a population with a low pre-test probability for systemic AID. ANA were determined in serum samples at baseline visit from 2997 participants from the Camargo Cohort using indirect immunofluorescence assay, and two solid phase assays (SPA), addressable laser bead immunoassay, and fluorescence enzyme immunoassay. Sociodemographic and biobehavioral features of the subjects were obtained at baseline visit using a structured questionnaire. The prevalence of ANA positive results was significantly higher when indirect immunofluorescence assay was used as screening method in comparison with SPAs, being higher in females, older subjects, and those with higher C-reactive protein levels. Considering biobehavioral features, the prevalence was higher in those individuals with a sedentary lifestyle, and in ex- and non-alcohol users. Moreover, considering the relevance of the antibody load using ANA Screen, the prevalence of the antibody load also increased with age, especially in females. In conclusion, the prevalence of ANA varies depending on sociodemographic and biobehavioral features of the subjects, which could be relevant specifically in a population with a low pre-test probability for systemic AIDs.

**Supplementary Information:**

The online version contains supplementary material available at 10.1007/s12026-023-09430-z.

## Introduction

Autoantibodies are the hallmark of autoimmunity, and specifically, antinuclear antibodies (ANA) are one of the most relevant antibodies present in systemic autoimmune diseases (AID). The presence of ANA, together with anti-dsDNA antibodies and extractable nuclear antigens (ENA), is important for the classification, diagnosis, and monitoring of patients with systemic AID. The first evidence of the existence of ANA was the description of the LE-cell phenomenon by Hargraves et al. in 1948 [1]. However, it was not until the early 1950s, due to the research performed by Coons, Kaplan, and Weller, when indirect immunofluorescence (IIF), using cryopreserved sections of rodent tissues as substrate, began to be used for the detection of ANA. Later on, in the mid-70s, it was discovered that human tissue culture cells, such as human epithelial type-2 cells (HEp-2 cells) derived from laryngeal carcinoma, were better than primary organ sections for the detection of ANA. This is so because the production of this type of cell in large numbers was easier and they had bigger nuclei and expressed antigens in various stages of the cellular cycle [2].

Despite being characteristic of systemic AID, ANA are not specific since they may be present in organ-specific AID [3], infections [4, 5], inflammatory disorders [6], neoplasms [7], or even in healthy individuals [8, 9].

Currently, the IIF assay using HEp-2 cells as substrate, due to its high sensitivity, continues to be one of the recommended methods for the study of ANA, as expressed in the position statement of the American College of Rheumatology (ACR), published in 2010, and also by the European League Against Rheumatism, EULAR in 2019 [10]. Likewise, as demonstrated in a recent publication by the Spanish group of autoimmunity (GEAI), it continues to be the most widely used method for ANA detection [11]. Nevertheless, in recent years, several publications have questioned this position and question whether IIF should be replaced by solid-phase assays (SPA) [12–16]. Despite all this technological development for the study of ANA, the 2019 EULAR/ACR classification criteria for SLE maintain the presence of ANAs using HEp-2 cells or an equivalent positive test as an entry criterion for SLE patients [17]. However, it should be noted that the IIF assay using HEp-2 cells is a laborious process, that needs skilled operators, and therefore, the reliability of the results is directly related to the experience of the technician.

In comparison with the IIF assay, SPAs are less sensitive but more specific for the detection of ANA in systemic AID [18, 19]. In addition, different studies have shown that combining the results obtained with both techniques (IIF and SPA) is more accurate than performing either of the two tests separately [14, 16, 20]. In fact, the single presence of ANA by IIF is not specific of AID, since these autoantibodies appear in up to 30% of healthy individuals, a proportion that increases with age, especially in women [8, 21–23].

However, all of this evidence generally comes from well-characterized cohorts of patients with systemic AIDs, and yet there are scarce data on the value of ANAs detected by IIF and SPA in unselected populations and especially in cohorts with a low pretest probability. This is a very important aspect to consider due to how the request for ANA has evolved over the years. Historically, only rheumatologists and clinical immunologists ordered ANA for diagnosis of systemic AID. Over time, several clinicians, such as nephrologists, internists, and gastroenterologists joined in requesting ANA, and currently, almost any clinician and even general practitioners should request them [24]. Initially, as a consequence of this increase in the number of requested tests, the pre-test probability for ANA detection was very high, and in recent years, it has been significantly reduced, reaching currently a very low pre-test probability. Therefore, due to this low pre-test probability, the post-test probability for ANA positivity is also low. Taking into account this aspect, we consider that it is of great importance to study the presence of ANA through IIF and SPA in a low pre-test probability population for systemic AID, such as the Camargo Cohort [25] since ANA detection by IIF may be better when the pre-test probability is high, whereas SPA techniques are more useful in populations with an overall low pre-test probability for systemic AID.

A less studied aspect is whether there is a relationship between the prevalence of ANA and the sociodemographic features of a population. Although some reports suggest a higher prevalence of ANA in females and older individuals [26, 27], the association between ANA and other sociodemographic factors is largely unknown. Therefore, in the present work, we evaluate the relationship between ANA and sociodemographic and biobehavioral factors in the Camargo Cohort, a well-established population with a low pre-test probability for systemic AID, which could reflect the current trend regarding the request for ANA.

## Methods

### Study population and serum collection

The Camargo Cohort study is a prospective community–based study designed to evaluate the prevalence and incidence of metabolic bone diseases and risk factors for osteoporosis and fragility fractures in Caucasian postmenopausal women and men older than 49 who attended a Primary Care Centre in Northern Spain for their regular health examination or medical reasons, whichever happened first. The study was set up between February 2006 and February 2011, and full details of this cohort have been previously reported [28–31].

In the present study, 2997 serum samples from all the subjects included in the Camargo Cohort have been analyzed. Specifically, the first available serum sample of each subject has been tested for the presence of ANA. This sample was selected to focus on an age range where age-associated increases in ANA become apparent with a low pretest probability.

The study was conducted according to the criteria *set* by the Declaration of Helsinki and was approved by the Regional Ethics Committee (CEIm Internal Code: 2020.309). All patients gave their written informed consent.

### Data collection and assessment of sociodemographic and biobehavioral features

At the baseline visit, subjects were interviewed by the investigators using a structured questionnaire that included age, sex, education level, smoking, alcohol consumption, physical exercise, cardiovascular risk factors, chronic disorders, body mass index (BMI), and serum C-reactive protein (CRP) levels. BMI was defined as the weight (kg) divided by height squared (m^2^), and was stratified as follows: underweight (<20), normal weight (20-25), overweight (25.01-29.99), and obese (≥30). Smoking was classified as current, ex-smoker, and non-smoker. Current alcohol intake was defined as >20 g of alcohol per day, and subjects were classified as current, ex-, and non-alcohol consumers. Physical activity was classified as sedentary (armchair to bed), moderate (<3 h/week of active exercise), and intense (> 3 h/week of active exercise).

### ANA screening methods

At baseline, serum samples from each subject were obtained and used for ANA testing. Specifically, ANA was determined by three different methods including IIF on HEp-2 cells (Biosystems, Barcelona, Spain), and two SPAs, such as ANA Screen (Bio-Rad, Hercules, CA, USA), and connective tissue disease (CTD) Screen (ThermoFisher, Freiburg, Germany).

### IIF assay on HEp-2 cells

Sera were diluted 1:160 with phosphate-buffered saline (PBS), which was considered the screening dilution. A cut-off 1:160 ANA titer was selected instead of 1:80 in order to obtain a high specificity (86.2% (CI 95% 80.4–90.5)) maintaining a relatively high sensitivity (95.8% (CI 95% 94.1–97.1)) [32]. A Zeiss fluorescence microscope with incident mercury light illumination and filters for activation/emission of fluorescein isothiocyanate (FITC) was used. Slides with fixed HEp-2 cells served as a source of nuclear antigens (Biosystems, Barcelona, Spain). FITC-conjugated rabbit anti-human IgG was used as the secondary antibody (Biosystems, Barcelona, Spain). Incubations, washing steps, and mounting microscope slides were done following the manufacturer’s instructions. The slides were inspected under the fluorescence microscope at 400 magnification. Nuclear, cytoplasmic, and mitotic HEp-2 patterns were considered, and the nomenclature for ANA detected using IIF assay on HEp-2 cells was performed according to the International Consensus on ANA Patterns (ICAP) [33, 34].

### ANA Screen Bio-Rad

The BioPlex 2200 ANA Screen system (Bio-Rad, Hercules [CA], USA) based on ALBIA technology was used to detect 13 types of autoantibodies simultaneously, such as those directed against dsDNA, chromatin, centromere B, Scl-70, RNP (RNP-A, RNP-68), Sm, RNP/Sm, Ro (SSA-52, SSA-60), SSB/La, Jo-1, and ribosomal P protein, following manufacturer’s instructions. The presence of anti-dsDNA antibody was classified as negative when levels were ≤4 IU/mL, indeterminate if 5 to 9 IU/mL, and positive when ≥10 IU/mL, as recommended by the manufacturer. For the other autoantibodies, the results were expressed as an antibody index (AI). An AI of 1.0 was the cut-off concentration that corresponded to approximately the 99th percentile of values obtained from a non-disease population in the manufacturer’s study. Results of ≥1.0 were reported as positive (range, 0.2–8.0 AI). A test result was considered positive for ANA if one or more of the antibody tests in the panel were positive.

### CTD Screen ThermoFisher

EliA CTD Screen (ThermoFisher, Freiburg, Germany) based on FEIA was run on Phadia 250 system (Thermo Fisher Scientific) to detect autoantibodies against 14 antigens, such as centromere (CENP-B), dsDNA, Jo-1, Mi-2, proliferating cell nuclear antigen (PCNA), polymyositis (PM)-Scl, ribosomal-P, RNA Pol III, Scl-70, Sm, SSA/Ro (Ro52 and Ro60), SSB/La, U1-RNP (RNP-70, A, and C), and fibrillarin, following manufacturer’s instructions. The results obtained were interpreted as positive, indeterminate, or negative according to the cut-off values specified by the manufacturer (>1.0 ratio, positive; 0.7–1.0 ratio, indeterminate; <0.7 ratio, negative). A test result was considered positive for ANA when one or more of the antibody tests in the panel were positive.

### Statistical analysis

The distribution of continuous variables was assessed using Kolmogorov–Smirnov or Shapiro–Wilk tests when indicated. Results were expressed as mean ± standard deviation for continuous variables and percentages for categorical data. Comparisons were based on the chi-squared test or Fisher test for categorical data and the Student’s *t*-test or Mann-Whitney *U*-test for parametric and non-parametric continuous variables, respectively. A two-tailed *p*-value <0.05 was considered significant in all the calculations. Statistical analysis was performed using SPSS 28.0 (IBM Corp., Armonk, NY, USA).

## Results

### Prevalence of ANA according to the three assays and association with sociodemographic characteristics

The sociodemographic and biobehavioral variables of the subjects included in the study are shown in Table 1. The prevalence of ANA positive results in the subjects of the Camargo Cohort using the three methods for ANA detection, considering the different sociodemographic features, is shown in Table 2.

**Table 1:** Demographic features of the study cohort

Variable	Total	Women	Men	*p*
Age, *yrs., mean ± SD*	64.7 ± 10.6	64.2 ± 11.2	65.5 ± 9.5	0.001
Sex, *n (%)*	2997	1941 (64.8)	1056 (35.2)	<0.001
Education level, *n (%)*				
*None*	43 (1.5)	32 (1.7)	11 (1.1)	0.24
*Primary*	2148 (73.9)	1419 (75.7)	729 (70.7)	0.02
*Secondary*	479 (16.5)	313 (16.7)	166 (16.1)	0.81
*Technical education*	106 (3.6)	43 (2.3)	63 (6.1)	<0.001
*University*	126 (4.3)	66 (3.5)	60 (5.8)	0.004
Exercise, *n (%)*				
*Sedentary*	155 (5.2)	122 (6.4)	33 (3.1)	<0.001
*Moderate*	1237 (41.6)	907 (47.3)	330 (31.4)	<0.001
*Intense*	1578 (53.1)	889 (46.4)	689 (65.5)	<0.001
Smoking, *n (%)*	442 (14.7)	243 (12.5)	199 (18.8)	<0.001
Diabetes mellitus, *n (%)*	437 (14.6)	227 (11.7)	210 (19.9)	<0.001
High blood pressure, *n (%)*	1364 (45.5)	846 (43.6)	517 (49.0)	0.005
Dyslipidemia, *n (%)*	917 (30.6)	554 (28.5)	363 (34.4)	0.001
BMI, *kg/m*^*2*^*, mean ± SD*	28.9±4.5	28.7±5.0	29.0±3.5	0.09
Alcohol intake, *n (%)*	741 (24.7)	226 (11.6)	515 (48.8)	<0.001
CRP, *mg/dl, median (IQR)*	0.20 (0.10-0.50)	0.20 (0.10-0.50)	0.20 (0.10-0.50)	0.09

*Abbreviations*: *BMI* body mass index, *CRP* serum C-reactive protein, *SD* standard deviation, *IQR* interquartile range

**Table 2:** Prevalence of antinuclear antibodies (ANA) according to the three assays and associations with selected sociodemographic features in Camargo Cohort subjects

Variable	ANA IIF + *n* = 2997	p*	ANA Screen + *n* = 2941	p*	CTD Screen + *n* = 2985	p*
Total, *n (%)*	774 (25.8)	-	400 (13.6)	-	235 (7.9)	-
Age (yrs.), *n (%)*		0.10		0.01		0.0001
*<50*	25 (30.1)		8 (10.0)		3 (3.7)	
*50–59*	266 (24.7)		119 (11.2)		52 (4.9)	
*60–69*	217 (24.1)		127 (14.3)		58 (6.5)	
*70–79*	171 (27.5)		94 (15.5)		78 (12.6)	
*≥80*	95 (30.0)		52 (16.8)		44 (13.9)	
Sex, *n (%)*		0.0001		0.003		0.003
*Male*	226 (21.4)		114 (11.0)		59 (5.6)	
*Female*	548 (28.2)		286 (15.0)		176 (9.1)	
Education level, *n (%)*		0.47		0.51		0.21
*None*	12 (27.9)		5 (11.9)		10 (23.3)	
*Elementary*	558 (26.0)		228 (13.7)		171 (8)	
*Secondary*	117 (24.4)		64 (13.6)		29 (6.1)	
*Vocational training*	26 (24.5)		14 (13.6)		6 (5.7)	
*University*	29 (23.0)		17 (13.5)		9 (7.1)	

*n*: reflects the number of subjects within the sample. %: reflects the percentage of subjects in each subgroup. *p for trend (excluding sex analysis) for each ANA technique

*Abbreviations*: *ANA* antinuclear antibodies, *IIF* indirect immunofluorescence

As previously stated, the prevalence of ANA positive results is significantly higher when IIF assay (25.8%) was used as screening method in comparison with SPAs, including ANA Screen (13.6%) and CTD Screen (7.9%) [25]. As it is known, the older the subject the higher the ANA prevalence, regardless of the method used, although this increase was more evident when SPAs (ANA Screen and CTD Screen) were used for the detection of those autoantibodies (*p* = 0.01 and *p* < 0.001, respectively) [9]. Likewise, independently of the methodology for ANA detection, the frequency of positive results was higher in women than in men. When education level was considered, no differences were observed in the prevalence of ANA. Nevertheless, using IIF or CTD Screen, a higher, albeit non-significant, frequency of ANA positive results was observed in the subgroup of subjects without studies.

### Prevalence of positive ANA and association with selected biobehavioral features

Several differences were observed in the prevalence of ANA according to the selected biobehavioral characteristics (Table 3). While no differences were observed in the prevalence of ANA according to the BMI, the prevalence of these autoantibodies varied depending on the physical activity and the alcohol intake of the subjects. Specifically, ANA-positive results were more frequent in participants with a sedentary lifestyle in comparison with individuals that performed moderate or intense physical activity. These results were observed when the screening of ANA was performed using IIF assay (*p* = 0.03) and CTD Screen (*p* = 0.01). Likewise, this trend was also observed using ANA Screen, without reaching a significant difference. Regarding alcohol consumption, independently of the method used for ANA detection, the prevalence of ANA was higher in ex- and non-alcohol consumers compared to current users. However, among the three methods used, only the CTD screen demonstrated to be significantly higher in both groups (*p* = 0.008). When the prevalence of ANA was analyzed regarding the smoking habit, a higher frequency of ANA positive results was observed in non-smoker subjects independently of the screening method used. Nevertheless, the observed differences did not reach statistical significance. Finally, using CTD Screen, a significant increase in the prevalence of ANA was found in those subjects with higher CRP levels (*p* = 0.01). This finding was also observed using IIF assay as a screening method for ANA detection.

**Table 3:** Estimated prevalence of antinuclear antibodies (ANA) in the Camargo Cohort according to BMI, smoking, alcohol intake, physical activity, and C-reactive protein

Variable	ANA IIF + *n* (%)	p*	ANA Screen + *n* (%)	p*	CTD Screen + *n* (%)	p*
BMI (*kg/m*^*2*^*)*, *n (%)*		0.98		0.90		0.52
*<20*	0 (0.0)		0 (0.0)		0 (0.0)	
*20–25*	146 (26.8)		72 (13.6)		43 (8.1)	
*25.01–29.99*	326 (25.0)		168 (13.1)		77 (5.9)	
*≥30*	263 (26.0)		137 (13.8)		91 (9.0)	
Smoking, *n (%)*		0.08		0.93		0.35
*Current*	118 (26.7)		54 (12.4)		21 (4.8)	
*Ex*	154 (22.7)		82 (12.4)		39 (5.8)	
*No*	502 (26.8)		264 (14.3)		175 (9.4)	
Alcohol intake, *n (%)*		0.11		0.06		0.008
*Current*	172 (23.2)		81 (11.1)		35 (4.7)	
*Ex*	62 (26.5)		34 (14.9)		22 (9.5)	
*No*	540 (26.7)		285 (14.4)		178 (8.8)	
Physical activity, *n (%)*		0.03		0.96		0.01
*Sedentary*	50 (32.3)		29 (19.2)		24 (15.5)	
*Moderate*	330 (26.7)		160 (13.1)		101 (8.2)	
*Intense*	387 (24.5)		207 (13.4)		109 (6.9)	
CRP (*mg/dl)*, *n (%)*		0.07		0.48		0.01
*<0.10*	236 (24.8)		105 (11.2)		51 (5.4)	
*0.10–0.49*	313 (25.5)		185 (15.3)		109 (8.9)	
*0.50–1.0*	106 (27.7)		48 (12.9)		30 (7.9)	
*>1.0*	70 (30.0)		32 (14.2)		26 (11.2)	

*n*: reflects the number of subjects within the sample. %: reflects the percentage of subjects in each subgroup. *p for trend for each ANA technique

*Abbreviations*: *ANA* antinuclear antibodies, *IIF* indirect immunofluorescence, *BMI* body mass index, *CRP* C-reactive protein

### Prevalence of ANA titers by IIF and associations with selected sociodemographic and biobehavioral features

The prevalence of ANA by IIF assay, considering the different antibody titers observed, showed different associations with the selected biobehavioral measures. Specifically, ANA positive results were considered as 1/160 or >1/160, and the specific titer obtained was also taken into account.

As can be seen in Supplementary Table 1, when the study population was stratified by age, as expected due to the low pretest probability of the subjects included, a high frequency of negative results was obtained at all ages, ranging from 69.9 to 75.9%. When ANA results were stratified in negative, positive titers 1/160 or >1/160 and analyzed across the age strata, the *p* for trend was 0.036.

Considering the prevalence of ANA based on sex, a significantly higher frequency of negative results was observed in males (*p* < 0.0001) (Table 4). Likewise, while no differences were observed in the frequency of 1/160 ANA positive results, a significant increase of titers >1/160 was observed in women (*p* < 0.0001). Specifically, females showed a significant increase of ANA-positive results at titers of 1/320 and >1/1280 compared to males (*p* < 0.0001, and *p* = 0.0002, respectively).

**Table 4:** Frequency of ANA positive results by indirect immunofluorescence (IIF) assay stratified by sex

	Male	Female	*p*
ANA IIF negative, *n* (%)	830 (78.6)	1392 (71.7)	<0.0001
ANA IIF + 1/160, *n* (%)	141 (13.4)	272 (14.0)	0.66
ANA IIF + >1/160, *n* (%)	85 (8.0)	277 (14.3)	<0.0001
*ANA IIF + 1/320, n (%)*	36 (3.4)	145 (7.5)	<0.0001
*ANA IIF + 1/640, n (%)*	29 (2.7)	41 (2.1)	0.33
*ANA IIF + 1/1280, n (%)*	14 (1.3)	40 (2.1)	0.19
*ANA IIF + >1/1280, n (%)*	6 (0.6)	49 (2.5)	0.0002

*n*: reflects the number of subjects within the sample. %: reflects the percentage of subjects in each subgroup

*Abbreviations*: *ANA* antinuclear antibodies, *IIF* indirect immunofluorescence

When the different ANA titers were considered according to the education level of the participants (Supplementary Table 2) or the smoking habit (data not shown), no differences were observed. Moreover, considering ANA titers and alcohol intake (Supplementary Table 3) or serum CRP quartiles (Supplementary Table 4), we found a trend for lower ANA titers in current drinkers (*p* = 0.059) and higher titers in participants in the higher quartiles of serum CRP levels (*p* = 0.058).

On the contrary, when the different titers of ANA were considered in subjects according to their physical activity, several differences were observed, as depicted in Table 5. Thus, while no differences were observed regarding 1/160 ANA positive results, the prevalence of ANA positive results at titers >1/160 was significantly higher in sedentary subjects in comparison with those who carried out some physical activity (p=0.0005).

**Table 5:** Frequency of ANA positive results by indirect immunofluorescence (IIF) assay stratified by physical activity

	Sedentary	Moderate	Intense	*p*
ANA IIF negative, *n* (%)	105 (67.7)	907 (73.3)	1190 (75.4)	0.07
ANA IIF + 1/160, *n* (%)	17 (11.0)	179 (14.5)	213 (13.5)	0.36
ANA IIF + >1/160, *n* (%)	33 (21.3)	151 (12.2)	175 (11.1)	0.0005
*ANA IIF + 1/320, n (%)*	17 (11.0)	70 (5.7)	94 (6.0)	0.01
*ANA IIF + 1/640, n (%)*	3 (1.9)	32 (2.6)	35 (2.2)	0.93
*ANA IIF + 1/1280, n (%)*	6 (3.9)	28 (2.3)	19 (1.2)	0.08
*ANA IIF + >1/1280, n (%)*	7 (4.6)	21 (1.7)	26 (1.6)	0.02

*n*: reflects the number of subjects within the sample. %: reflects the percentage of subjects in each subgroup. *p* values represent the differences between sedentary subjects and those who perform any physical activity. *p*-value for trend = 0.002 for each exercise category

*Abbreviations*: *ANA* antinuclear antibodies, *IIF* indirect immunofluorescence

### Relevance of the antibody load and associations with sociodemographic and biobehavioral characteristics

The comparison between the antibody load observed in Camargo Cohort subjects using ANA Screen, which gives information about 14 specificities, and the selected sociodemographic features and biobehavioral measures is shown in Table 6. According to the number of autoantibodies, the subjects were classified as follows: antibody load 0 (no autoantibody, 86.4%), antibody load 1 (1 autoantibody, 12.4%), and antibody load ≥2 (2 or more autoantibodies, 1.2%). An increasing frequency of antibody loads 1 and ≥2 was detected as the age of the subjects increased (*p* = 0.001).

**Table 6:** Antibody load using ANA Screen assay according to selected sociodemographic features and biobehavioral characteristics of Camargo Cohort subjects

	Antibody load 0	Antibody load 1	Antibody load ≥2	p*
Total, *n (%)*	2541 (86.4)	364 (12.4)	36 (1.2)	-
Age (yrs.), *n (%)*				0.001
*<50*	72 (90.0)	6 (7.5)	2 (2.5)	
*50–59*	939 (88.8)	112 (10.6)	7 (0.7)	
*60–69*	759 (85.7)	117 (13.2)	10 (1.1)	
*70–79*	513 (84.5)	83 (13.7)	11 (1.8)	
*≥80*	258 (83.2)	46 (14.8)	6 (1.9)	
Sex, *n (%)*				0.0001
*Male*	923 (89.0)	110 (10.6)	4 (0.4)	
*Female*	1618 (85.0)	254 (13.3)	32 (1.4)	
Education level, *n (%)*				0.56
*None*	37 (88.1)	4 (9.5)	1 (2.4)	
*Elementary*	1816 (86.3)	258 (12.3)	30 (1.4)	
*Secondary*	408 (86.4)	62 (13.1)	2 (0.4)	
*Vocational training*	89 (86.4)	14 (13.6)	0 (0.0)	
*University*	109 (86.5)	15 (11.9)	2 (1.6)	
BMI (*kg/m*^*2*^*), n (%)*				0.68
*<20*	4 (100)	0 (0.0)	0 (0.0)	
*20–25*	456 (86.4)	66 (12.5)	6 (1.1)	
*25.01–29.99*	1111 (86.9)	153 (12.0)	15 (1.2)	
*≥30*	854 (86.2)	123 (12.4)	14 (1.2)	
Smoking, *n (%)*				0.62
*Current*	382 (87.6)	49 (11.2)	5 (1.1)	
*Ex-smoker*	579 (87.6)	78 (11.8)	4 (0.6)	
*Non-smoker*	1580 (85.7)	237 (12.9)	27 (1.5)	
Alcohol intake, *n (%)*				0.08
*Current*	646 (88.9)	74 (10.2)	7 (1.0)	
*Ex*	194 (85.1)	34 (14.9)	0 (0.0)	
*No*	1701 (85.6)	256 (12.9)	29 (1.5)	
Physical activity, *n (%)*				0.17
*Sedentary*	122 (80.8)	26 (17.2)	3 (2.0)	
*Moderate*	1058 (86.9)	142 (11.7)	18 (1.5)	
*Intense*	1339 (86.6)	193 (12.5)	14 (0.9)	
CRP *(mg/dl)*, *n (%)*				0.11
*<0.10*	835 (88.8)	96 (10.2)	9 (1.0)	
*0.10–0.49*	1025 (84.7)	170 (14.0)	15 (1.2)	
*0.50–1.0*	324 (87.1)	40 (11.8)	8 (2.2)	
*>1.0*	193 (85.8)	29 (12.9)	3 (1.3)	

*n*: reflects the number of subjects within the sample. %: reflects the percentage of subjects in each subgroup. “*p*” represents p-values for trend, except for sex (for each antibody load category)

*Abbreviations*: *BMI* body mass index, *CRP* C-reactive protein

Once again, when the sex of the participants was considered, a significantly higher frequency of positive results (antibody load 1 and ≥2) was observed in females. Regarding alcohol intake, the antibody load tended to be lower in current drinkers (*p* = 0.08). No significant differences were observed in the different sociodemographic features and biobehavioral measures in terms of antibody load.

## Discussion

The determination of ANA is very important for the diagnosis of patients with suspected systemic AID. However, less is known about the true value of these autoantibodies in populations with a low pre-test probability for systemic AID, and even less about their association with sociodemographic features in these populations.

The present study provides an estimation of the prevalence of ANA, using an IIF assay and two different SPAs, according to different sociodemographic factors and with selected biobehavioral measures. Our results show an overall prevalence of ANA of 25.8% by IIF at 1/160 serum-dilution, 13.6% using ANA Screen, and 7.9% using CTD Screen, which is higher in the case of IIF in comparison to other published reports in healthy selected populations [35–37]. These differences likely relate to the characteristic of the different study populations and the variations in ANA assessment across laboratories. The higher prevalence of ANA detected by IIF assay using HEp-2 cells, in comparison with SPA, could be due to the fact that HEp-2 cells have approximately 100–150 possible autoantigens, which can be considered the bigger array of autoantigens, while not all specific autoantigens are included in SPAs. Likewise, independently of the methodology used for ANA detection, the frequency of positive results was higher in women than in men. Besides, considering the age of the participants, the older the subject the higher the prevalence of ANA, regardless of the method, although this increase was more evident when SPAs (ANA Screen and CTD Screen) were used [22, 38, 39]. This linear tendency was not observed using IIF, probably due to the presence of ANA patterns that are not associated with any detectable antigenic specificity, as reported in some studies [40]. Furthermore, in line with the results published by other authors, ANA prevalence did not vary with the education level [35].

Considering the association between ANA with selected biobehavioral variables, unlike other studies in which it was observed that the presence of ANA is less common in overweight and obese individuals than in normal-weight individuals [35], we did not observe differences in the prevalence of ANA according to the BMI categories. However, the prevalence of these autoantibodies varied depending on the physical activity and the alcohol intake of the participants. Thus, the prevalence was higher in those individuals with a sedentary lifestyle and in ex- and non-alcohol users compared to current alcohol consumers. The results related to physical activity could be important because the presence of ANA has been associated with decreased carotid elasticity, suggesting that mechanisms resulting in ANA production may be involved in the development of early atherosclerosis [41]. Other authors have also shown that moderate alcohol consumption has been associated with decreased systemic lupus erythematosus (SLE) risk [42]. Likewise, it has also been described in several studies that alcohol acts as a protective factor against the development of other AID, such as diabetes [43], multiple sclerosis, [44] or especially, rheumatoid arthritis (RA) [45, 46]. In the case of RA, upon alcohol exposure, different factors have been identified to be reduced, such as antigen-presentation, T cell activation capacity of antigen-presenting cells, B cell maturation and proliferation, IL-21 production by T follicular helper cells, antigen-specific IgG, and proinflammatory cytokines. Moreover, the Th2 immune response, M2 macrophage function, and anti-inflammatory cytokines (IL-10, TGF-β) [47] seem to be increased. All these factors could be associated with a reduced risk of RA. In the present study, we observed an increasing, albeit non-significant, trend in the antibody load in non-alcohol consumers compared to current drinkers.

In addition, despite some studies suggesting smoking as a risk factor for SLE, RA, and other AID [48], we found a higher frequency of ANA-positive results in non-smoker subjects independently of the screening method used. Finally, using IIF and CTD Screen, a significant increase in the prevalence of ANA was found in those subjects with higher serum CRP levels, which could indicate the involvement of systemic inflammation in the development of systemic AID. In the same way, this finding could be related to the processes of immunosenescence and autoimmunity that are observed in aging people, in whom, the development of autoreactive T cells, as well as an increase in the production of autoantibodies, has been described [49–51]. Moreover, there is a relationship between immunosenescence and the development of a senescence-associated secretory phenotype (SASP), which has been associated with malignancy progression as well as with autoimmune disorders [51–54].

One could speculate that our findings regarding the association of ANA with different sociodemographic and behavioral factors might be related to the sample size or even by chance. However, in our study, the greater the specificity of the test [55], the greater the statistical significance of some relevant sociodemographic variables analyzed, such as age, alcohol intake, physical activity, and serum CRP levels (as a subrogate marker of inflammation). Due to the scarcity of data and these potential associations, further studies are needed to confirm our findings.

To our knowledge, this is the first study in which the relevance of the autoantibody load is considered, as has been described in antiphospholipid syndrome (APS). In the APS, depending on the number, the type, and the titer of antiphospholipid antibodies, it is possible to establish different risk profiles [55]. In our study, focusing on the relevance of the antibody load using ANA Screen and their associations with selected sociodemographic features and biobehavioral measures showed that among subjects with antibody loads 1 (one autoantibody) and ≥2 (two or more autoantibodies), in addition to the prevalence of ANA, the prevalence of the antibody load also increases with age.

Our study has some limitations. Firstly, those inherent to a cross-sectional study. Secondly. the evaluation of antigenic specificities in those patients with ANA positive results in the screening assay using SPA was only possible using the ANA screen and not the CTD screen. Thirdly, not all types of autoantibodies were assessed. Finally, the small simple size of certain subgroups may have limited our power to detect differences in ANA prevalence for some factors. Nevertheless, our study also has several strengths, such as the large number of subjects included belonging to a well-established population cohort with a low pre-test probability for systemic AID, and a long-term follow-up, which could reflect the current trend regarding the request for ANA and allow us to draw more robust conclusions. There is no other study in which so many subjects have been included, with such a long follow-up period, where the association between the presence of ANA and selected sociodemographic features and biobehavioral measures had been evaluated.

In conclusion, the prevalence of ANA is higher using IIF assay than SPA in a population-based cohort with a low pretest probability for AIDs. However, independently of the technique used, the prevalence of ANA is higher in females and older subjects. Considering biobehavioral measures, ANA-positive results were frequent in those individuals with a sedentary lifestyle, as well as in ex-and non-alcohol consumers. Finally, it is important to highlight the study of the antibody load, since it may be relevant when establishing the risk profile of population-based subjects.

### Supplementary information

ESM 1 (DOCX 19 kb)
