# Supplementary material for: Prevalence and sociodemographic correlates of antinuclear antibody testing by indirect immunofluorescence or solid-phase assays in a Spanish population: the Camargo Cohort
Source: Immunol Res. 2023 Nov 4;72(2):260–70. doi: 10.1007/s12026-023-09430-z (PMC11031476; doi:10.1007/s12026-023-09430-z)
Supplement: Supplementary file 1 — (DOCX 19 kb) [file 12026_2023_9430_MOESM1_ESM.docx]

**Supplementary Table 1.** Frequency of ANA positive results by indirect immunofluorescence (IIF) assay stratified by age.

|  | <50 yrs. | 50-59 yrs. | 60-69 yrs. | 70-79 yrs. | ≥80 yrs. |
| --- | --- | --- | --- | --- | --- |
| ANA IIF negative, *n (%)* | 58 (69.9) | 808 (75.2) | 683 (75.9) | 451 (72.5) | 222 (70.0) |
| ANA IIF + 1/160, *n (%)* | 11 (13.3) | 149 (13.9) | 128 (14.2) | 80 (12.9) | 45 (14.2) |
| ANA IIF + >1/160, *n (%)* | 14 (16.9) | 118 (11.0) | 89 (9.9) | 91 (14.6) | 50 (15.8) |
| *ANA IIF + 1/320, n (%)* | 7 (8.4) | 67 (6.2) | 38 (4.2) | 42 (6.8) | 27 (8.5) |
| *ANA IIF + 1/640, n (%)* | 2 (2.4) | 19 (1.8) | 19 (2.1) | 21 (3.4) | 9 (2.8) |
| *ANA IIF + 1/1280, n (%)* | 2 (2.4) | 14 (1.3) | 19 (2.1) | 13 (2.1) | 6 (1.9) |
| *ANA IIF + >1/1280, n (%)* | 3 (3.6) | 17 (1.6) | 12 (1.3) | 15 (2.4) | 8 (2.5) |

n: reflects the number of subjects within the sample. %: reflects the percentage of subjects in each subgroup. *p-value for trend=0.02

Abbreviations: ANA: antinuclear antibodies; IIF: indirect immunofluorescence

**Supplementary Table 2.** Frequency of ANA positive results by indirect immunofluorescence (IIF) assay stratified by education level.

|  | None | Elementary | Secondary | Vocational training | University |
| --- | --- | --- | --- | --- | --- |
| ANA IIF negative, n (%) | 31 (72.1) | 1590 (74.0) | 361 (75.4) | 80 (75.5) | 97 (77.0) |
| ANA IIF + 1/160, n (%) | 4 (9.3) | 300 (14.0) | 59 (12.3) | 16 (15.1) | 18 (14.3) |
| ANA IIF + >1/160, n (%) | 8 (18.6) | 258 (12.0) | 59 (12.3) | 10 (9.4) | 11 (8.7) |
| *ANA IIF + 1/320, n (%)* | 5 (11.6) | 129 (6.0) | 29 (6.1) | 4 (3.8) | 7 (5.6) |
| *ANA IIF + 1/640, n (%)* | 1 (2.3) | 49 (2.3) | 12 (2.5) | 4 (3.8) | 2 (1.6) |
| *ANA IIF + 1/1280, n (%)* | 0 (0.0) | 44 (2.0) | 6 (1.3) | 0 (0.0) | 0 (0.0) |
| *ANA IIF + >1/1280, n (%)* | 2 (4.7) | 35 (1.6) | 11 (2.3) | 2 (1.9) | 2 (1.6) |

n: reflects the number of subjects within the sample. %: reflects the percentage of subjects in each subgroup. *p-value for trend=0.59

Abbreviations: ANA: antinuclear antibodies; IIF: indirect immunofluorescence

**Supplementary Table 3.** Frequency of ANA positive results by indirect immunofluorescence (IIF) assay stratified by alcohol intake.

|  | Current | Ex | No |
| --- | --- | --- | --- |
| ANA IIF negative, n (%) | 569 (76.8) | 172 (73.5) | 1482 (73.3) |
| ANA IIF + 1/160, n (%) | 98 (13.2) | 36 (15.4) | 280 (13.8) |
| ANA IIF + >1/160, n (%) | 74 (10.1) | 26 (11.1) | 260 (12.9) |
| *ANA IIF + 1/320, n (%)* | 34 (4.6) | 12 (5.1) | 135 (6.7) |
| *ANA IIF + 1/640, n (%)* | 24 (3.2) | 3 (1.3) | 43 (2.1) |
| *ANA IIF + 1/1280, n (%)* | 9 (1.2) | 6 (2.6) | 39 (1.9) |
| *ANA IIF + >1/1280, n (%)* | 7 (0.9) | 5 (2.1) | 43 (2.1) |

n: reflects the number of subjects within the sample. %: reflects the percentage of subjects in each subgroup. *p-value for trend=0.059

Abbreviations: ANA: antinuclear antibodies; IIF: indirect immunofluorescence

**Supplementary Table 4.** Frequency of ANA positive results by indirect immunofluorescence (IIF) assay stratified by serum C-reactive protein quartiles (mg/dl).

|  | <0.10 | 0.10-0.49 | 0.50-1.0 | >1.0 |
| --- | --- | --- | --- | --- |
| ANA IIF negative, *n (%)* | 717 (75.2) | 915 (74.5) | 276 (72.3) | 163 (70.0) |
| ANA IIF + 1/160, *n (%)* | 133 (14.0) | 168 (13.7) | 49 (12.8) | 39 (16.7) |
| ANA IIF+ >1/160, *n (%)* | 105 (11.0) | 145 (11.8) | 57 (14.9) | 31 (13.3) |
| *ANA IIF + 1/320, n (%)* | 52 (5.5) | 76 (6.2) | 29 (7.6) | 14 (6.0) |
| *ANA IIF + 1/640, n (%)* | 18 (1.9) | 30 (2.4) | 13 (3.4) | 4 (1.7) |
| *ANA IIF + 1/1280, n (%)* | 15 (1.6) | 18 (1.5) | 10 (2.6) | 8 (3.4) |
| *ANA IIF + >1/1280, n (%)* | 18 (1.9) | 21 (1.7) | 5 (1.3) | 5 (2.1) |

n: reflects the number of subjects within the sample. %: reflects the percentage of subjects in each subgroup. *p-value for trend=0.058

Abbreviations: ANA: antinuclear antibodies; IIF: indirect immunofluorescence
